# Long-Term Outcomes of Elective Endovascular Aneurysm Repair for Abdominal Aortic Aneurysm in Japanese Elderly Patients

**DOI:** 10.5761/atcs.oa.24-00185

**Published:** 2025-01-21

**Authors:** Toshiya Nishibe, Masaki Kano, Shinobu Akiyama, Toru Iwahashi, Shoji Fukuda

**Affiliations:** 1 Department of Medical Informatics and Management, Hokkaido Information University, Ebetsu, Hokkaido, Japan; 2 Department of Cardiovascular Surgery, Tokyo Medical University, Tokyo, Japan

**Keywords:** endovascular aneurysm repair, abdominal aortic aneurysm, elderly, mortality

## Abstract

**Purpose:** Our primary concern was the risk of overtreating elderly patients with endovascular aneurysm repair (EVAR) for abdominal aortic aneurysm. We investigated the association between age at the time of EVAR and all-cause mortality in Japan’s aging population by stratifying patients into age groups.

**Methods:** Data from 175 patients who underwent elective EVAR from 2012 to 2016 were analyzed. Patients were categorized into 3 age groups: <75 years, 75–84 years, and ≥85 years, based on Japan’s healthy life expectancy and average life expectancy. Survival rates and risk factors for mortality were assessed across these patient groups.

**Results:** Among 175 patients, 3- and 5-year survival rates were significantly lower in elderly patients, with rates of 74.6% and 64.2% for those aged 75–84 years and 51.9% and 39.7% for those aged ≥85 years. Multivariate analysis identified age ≥85 years, chronic kidney disease, chronic obstructive pulmonary disease, and active cancer as independent adverse predictors of all-cause mortality, whereas obesity was identified as an independent protective predictor.

**Conclusions:** Adjusting guidelines to incorporate not only comorbidities but also age could optimize outcomes and healthcare resource allocation by prioritizing EVAR for patients most likely to benefit in Japan’s super-aging society.

## Introduction

Abdominal aortic aneurysm (AAA) is rare in individuals under 50 years of age, but its prevalence increases rapidly with age.^1)^ The global population is aging rapidly, with Japan at the forefront. The number of people aged ≥80 years in Japan was 12.2 million (10.3%) in 2024, and this figure is projected to reach 14.0 million (15.2%) by 2050.^2)^ The prevalence of AAA is increasing over time and therefore the management of AAA is critical in a super-aging society such as Japan.

Elective endovascular aneurysm repair (EVAR) is recognized as a minimally invasive and effective intervention; however, elderly patients, particularly octogenarians, are not often included in randomized controlled trials.^3)^ Nevertheless, elective EVAR for octogenarians is frequently performed in clinical practice, but surprisingly, there are limited long-term data available to evaluate the safety and outcomes of EVAR in this population.^4)^ Given the high prevalence of comorbidities and reduced physiologic reserve in elderly patients, the routine use of EVAR in this group warrants further investigation.^5)^

The purpose of this study was to assess whether there is an association between age at the time of EVAR and all-cause mortality in Japan’s aging population, stratified by age group. Considering that the life expectancy and healthy life expectancy of Japanese individuals were 84.3 and 74.1 years of age in 2019,^6)^ respectively, we compared all-cause mortality following EVAR in patients aged <75 years, 75–84 years, and ≥85 years.

## Materials and Methods

### Study design and patient characteristics

Data were retrospectively analyzed from a prospectively maintained database at Tokyo Medical University Hospital for patients who underwent elective EVAR for AAA between March 2012 and December 2016. The indications for EVAR comprised symptomatic AAA, asymptomatic AAA ≥5 cm in diameter, or rapidly expanding AAA (5 mm in 6 months) based on the Guideline for the Diagnosis and Treatment of Aortic Aneurysm and Aortic Dissection issued by Japanese Circulation Society.^7)^ Smaller AAAs (<5 cm) in conjunction with common iliac artery aneurysm ≥3 cm in diameter or if the patient strongly requested treatment were also included. Patients who were treated for ruptured aneurysms, infected aneurysms, residual aneurysms, and anastomotic pseudoaneurysms were excluded. Patients were divided into the following age groups: <75 years, 75–84 years, and ≥85 years, based on the life expectancy and healthy life expectancy of Japanese individuals.

At the initial consultation, all the patients were asked whether they were willing to provide written informed consent for their clinical data to be used for scientific presentations or publications. The procedures followed were in accordance with the Ethical Guidelines for Medical and Biological Research Involving Human Subjects (enacted on 23 March 2021 by the Japanese Government) and the Helsinki Declaration of 1975, as revised in 2008. This study was approved by the clinical research committee of Tokyo Medical University, where it was performed (TS2020-0388, January 15, 2021).

### Clinical risk factors

Baseline data on demographic and clinical risk factors: age, sex, smoking status (current, past), obesity (body mass index [BMI] ≥25 kg/m^2^), comorbidities (hypertension, dyslipidemia, diabetes, chronic kidney disease [CKD, defined as estimated glomerular filtration rate <60 mL/min/1.73 m^2^], chronic obstructive pulmonary disease [COPD], cerebrovascular disease, ischemic heart disease, active cancer), medications (antiplatelet agents [aspirin and/or clopidogrel], statins, β-blockers), aneurysm diameter, operative details (technical success, internal iliac artery embolization, outside of instruction for use [IFU], operative endoleaks), and secondary interventions were retrieved from the database. Secondary interventions were performed in patients with stent graft leg stenosis or occlusion, stent graft infection, type I or type III endoleaks, and type II endoleaks with aneurysm sac enlargement (>5–10 mm).

### Mortality

Patients were followed through the database until death or the end of the follow-up period in January 2021, as per institutional protocol.^8,9)^ Survival status was confirmed via outpatient visits, letters, or phone calls when clinic visits were not possible.

### Statistical analysis

Statistical analyses were performed using PRISM 9 for MAC OS X (GraphPad Software, Inc., La Jolla, CA, USA) and Mac Toukeikaiseki (Esumi, Tokyo, Japan). Data are presented as means ± standard deviations. Categorical data were compared using the χ^2^ test, and continuous data were analyzed using analysis of variance. The Kaplan–Meier method was used to evaluate time-to-event data among the groups, with comparisons of Kaplan–Meier curves performed using the log-rank test. To adjust for multiple comparisons, the Bonferroni method or Benjamini–Hochberg procedure was applied while setting the level of significance. Cox proportional hazard analyses were performed to identify factors that are independently associated with poor overall survival; variables with *p* <0.05 were entered into a multivariate model.

## Results

### Patients characteristics

The cohort comprised 175 patients (147 men and 28 women), with a median age of 79 years: 36 patients aged ≥85 years (86.9 ± 1.3 years), 76 patients aged 75–84 years (80.0 ± 2.6 years), and 63 patients aged <75 years (69.0 ± 5.1 years). Data by group are shown in **Table 1**. There were significant differences in the prevalence of obesity, past smoking, CKD, COPD, and small aneurysms among patients aged ≥85 years, 75–84 years, and <75 years. Post hoc tests revealed that obesity, past smoking, and CKD were significantly more or less prevalent in patients aged ≥85 years compared to those aged <75 years (*p* = 0.004, *p* <0.001, and *p* <0.001, respectively); COPD was significantly less common in patients aged <75 years than in patients aged 75–84 years (*p* = 0.014); small aneurysms were significantly less often indicated in patients aged <75 years compared to those aged 75–84 years and ≥85 years (*p* = 0.003 and *p* = 0.006, respectively).

**Table 1:** Patient characteristics

Variables	≥85 years (n = 36)	75–84 years (n = 76)	<75 years (n = 63)	*p*-value
Age, year	86.9 ± 1.3	80.0 ± 2.6	69.0 ± 5.1	<0.001*
Sex				
Female	8 (22.2)	12 (15.8)	8 (12.7)	0.461
BMI, kg/m^2^	21.7 ± 3.2	22.5 ± 3.6	23.8 ± 3.2	0.008*
Obesity (BMI >25 kg/m^2^)	3 (8.3)	18 (23.7)	22 (34.9)	0.012*
Smoking status				
Current	3 (8.3)	16 (21.1)	18 (28.6)	0.060
Past	16 (44.4)	46 (60.5)	49 (77.8)	0.034*
Comorbidities				
Hypertension	31 (86.1)	61 (80.3)	44 (69.8)	0.135
Dyslipidemia	17 (47.2)	35 (46.1)	27 (42.9)	0.895
Diabetes	9 (25.0)	16 (21.1)	13 (20.6)	0.864
CKD	24 (66.7)	36 (47.4)	20 (31.7)	0.003*
COPD	7 (19.4)	29 (38.2)	12 (19.0)	0.021*
Cerebrovascular disease	14 (38.9)	30 (39.5)	15 (23.8)	0.115
Ischemic heart disease	19 (52.8)	43 (56.6)	27 (42.9)	0.264
Active cancer	13 (36.1)	18 (23.7)	16 (25,4)	0.363
Medication				
Antiplatelet drugs	10 (27.8)	37 (48.7)	22 (34.9)	0.070
Statins	15 (41.7)	35 (46.1)	25 (39.7)	0.742
β-blockers	16 (44.4)	22 (28.9)	25 (39.7)	0.210
Aneurysm diameter, mm	55.0 ± 7.5	51.3 ± 7.0	52.4 ± 8.9	0.066
Small aneurysms, <55 mm	15 (41.7)	54 (71.1)	44 (69.8)	0.006*
Operative data				
Outside of IFU	8 (22.2)	13 (17.1)	8 (12.7)	0.465
Technical success	36 (100)	76 (100)	62 (98.4)	0.409
Internal iliac artery embolization	2 (5.6)	10 (13.2)	7 (11.1)	0.481
Operative endoleak				
Type I	0 (0)	0 (0)	0 (0)	N/A
Type II	3 (8.3)	20 (26.3)	15 (23.8)	0.086
Type III	0 (0)	0 (0)	1 (1.6)	0.409
Type IV	3 (8.3)	11 (14.5)	4 (6.3)	0.266
Secondary interventions	5 (13.9)	16 (21.1)	12 (19.0)	0.663
Mean follow-up period	3.25 ± 2.56	4.51 ± 2.32	5.94 ± 1.88	<0.001*

Unless indicated otherwise, data are presented as mean ± SD or n (%); *Significant.

BMI: body mass index; CKD: chronic kidney disease; COPD: chronic obstructive pulmonary disease; IFU: instruction for use

### All-cause mortality

During the study period, 61 patients (34.9%) died and 18 (10.3%) were lost to follow-up, resulting in a follow-up rate of 89.7%. The most common cause of death was cancer, followed by circulatory (cerebrovascular, cardiac, and renal) or pulmonary disease in all patient groups (**Table 2**). Two patients died of ruptured aneurysms or infection in patients aged ≥85 years and those aged 75-84 years, respectively.

**Table 2:** Causes of death by age groups

	≥85 years	75–84 years	<75 years
Cancer	9 (40.9)	10 (33.3)	6 (66.7)
Circulatory disease	5 (22.7)	8 (24.2)	2 (22.2)
Cerebrovascular	0 (0)	3 (10.0)	2 (22.2)
Cardiac	4 (18.2)	1 (3.3)	0 (0)
Renal	1 (4.5)	4 (13.3)	0 (0)
Pulmonary disease	3 (13.6)	10 (33.3)	1 (11.1)
Aneurysm-related death	1 (4.5)	1 (3.3)	0 (0)
Others	4 (18.2)	1 (3.3)	0 (0)
Total	22	30	9

Data are presented as n (%).

**Fig. 1:**
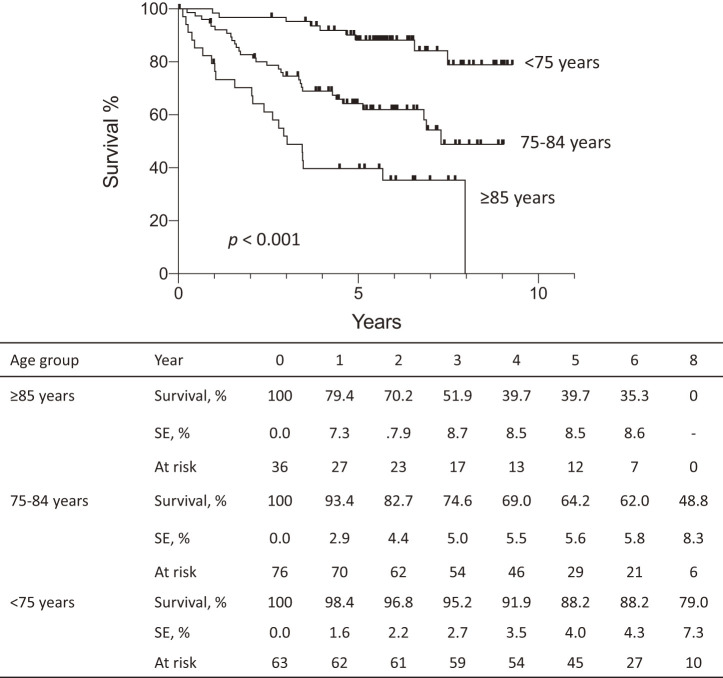
Kaplan–Meier curves of overall survival following endovascular aneurysm repair of patients aged <75 years, 75–84 years, and ≥85 years. Overall survival is significantly lower in patients aged ≥85 years compared to those aged 75–84 years and <75 years, and it was also significantly lower in patients aged 75–84 years than those aged <75 years (all *p* <0.001). SE, standard error

The median follow-up period was 4.97 years (interquartile range, 2.99–6.56 years), with a significantly shorter follow-up duration in patients aged ≥85 years compared to those aged 75–84 years and <75 years (both *p* <0.001, **Table 1**). Kaplan–Meier analysis showed that overall survival was significantly lower in patients aged ≥85 years compared to those aged 75–84 years and <75 years, and it was also significantly lower in patients aged 75–84 years than those aged <75 years (all *p* <0.001, **Fig. 1**). Median survival time was 3.02 years in patients aged ≥85 years and 7.30 years in those aged 75–84 years, while it could not be estimated in those aged <75 years due to the shorter follow-up period (≤9.28 years).

### Univariate and multivariate analyses for all-cause mortality

**Table 3** presents the results of univariate and multivariate analyses of risk factors affecting all-cause mortality. Univariate analysis found potential risk factors associated with poorer overall survival: age ≥85 years (vs. age 75–84 years and <75 years, *p* <0.001), obesity (*p* = 0.005), CKD (*p* = 0.019), COPD (*p* <0.001), active cancer (*p* = 0.001), and type IV endoleak (*p* = 0.007). Multivariate analysis further confirmed that age ≥85 years (vs. ages 75–84 and <75 years; hazard ratio [HR]: 3.47; confidence interval [CI]: 1.92–6.28; *p* <0.001), CKD (HR: 1.71; CI: 1.01–2.92; *p* = 0.048), COPD (HR: 2.07; CI: 1.18–2.63; *p* = 0.011), and active cancer (HR: 2.57; CI: 1.50–4.41; *p* = 0.001) were independent adverse predictors of all-cause mortality, whereas obesity (HR: 0.35; CI: 0.16–0.74; *p* = 0.006) was identified as an independent protective predictor.

**Table 3:** Risk factors affecting all-cause mortality

Variables	Univariate		Multivariate	
	HR (95% CI)	*p*-value	HR (95% CI)	*p*-value
Age				
≥85 years vs. 75–84 years and <75 years	3.41 (2.02–5.78)	<0.001*	3.47 (1.92–6.28)	<0.001*
Sex				
Female	0.59 (0.25–1.37)	0.219		
Obesity				
BMI >25.0	0.34 (0.16–0.72)	0.005*	0.35 (0.16–0.74)	0.006*
Smoking status				
Current	0.93 (0.50–1.72)	0.821		
Past	0.82 (0.49–1.37)	0.455		
Comorbidity				
Hypertension	1.25 (0.66–2.35)	0.495		
Dyslipidemia	0.78 (0.47–1.30)	0.342		
Diabetes mellitus	1.07 (0.59–1.94)	0.821		
CKD	1.83 (1.10–3.05)	0.019*	1.71 (1.01–2.92)	0.048*
COPD	3.04 (1.70–5.43)	<0.001*	2.07 (1.18–3.63)	0.011*
Cerebrovascular disease	1.33 (0.79–2.25)	0.460		
Coronary artery disease	1.10 (0.66–1.82)	0.716		
Active cancer	2.32 (1.39–3.87)	0.001*	2.57 (1.50–4.41)	0.001*
Medication				
Antiplatelet drugs	0.82 (0.48–1.38)	0.452		
Statins	0.78 (0.47–1.31)	0.345		
β-blockers	0.93 (0.55–1.58)	0.797		
Aneurysm morphology				
Small aneurysm, <55 mm	0.71 (0.43–1.18)	0.190		
Operative data				
Outside of IFU	0.82 (0.39–1.72)	0.594		
Internal iliac artery embolization	1.59 (0.79–3.25)	0.196		
Operative endoleak				
Type II	0.52 (0.25–1.10)	0.085		
Type IV	2.39 (1.26–4.52)	0.007*	1.89 (0.97–3.68)	0.063
Secondary interventions	0.62 (0.29–1.31)	0.209		

*Significant.

HR: hazard ratio; CI: confidence interval; BMI: body mass index; CKD: chronic kidney disease; COPD: chronic obstructive pulmonary disease; IFU: instruction for use

## Discussion

This study provides crucial insight into the long-term prognosis of elective EVAR for AAA in elderly patients in Japan’s super-aging society. Our findings indicate a significant decline in overall survival among patients aged ≥85 years and 75–84 years, with 3- and 5-year survival rates significantly lower than those observed in patients aged <75 years. The reduced long-term survival in patients aged ≥85 years and 75–84 years suggests that, while EVAR is reported to be less invasive and offers better perioperative outcomes than open surgery,^3)^ the long-term benefits may be diminished in this population.

In Japan’s super-aging society, where the population over 80 years is expected to nearly triple by 2050, it is becoming increasingly important to properly manage AAA in the elderly. This study was based on Japanese life expectancy data and classified patients into three age groups: <75 years, 75–84 years (between healthy life expectancy and life expectancy), and ≥85 years; this stratification reflects Japan’s average life expectancy of 84.3 years and healthy life expectancy of 74.1 years,^6)^ offering a more refined approach to assessing outcomes by aligning patients’ life stages and potential health trajectories with treatment decisions.

Consistent with those of Vaughan-Burleigh et al.,^5)^ our results show a significant decline in long-term survival in patients aged 75–84 years and ≥85 years; we found that the 3- and 5-year survival rates for patients aged 75–84 years and ≥85 years were 74.6% and 64.2% or 51.9% and 39.7%, respectively, compared with much higher rates for patients aged <75 years (95.2% and 88.2%). These results suggest that patients with a healthy life expectancy or less (<75 years) may be generally most likely to benefit from EVAR probably because of their higher physiologic reserve, whereas a more selective approach may be justified for patients near or exceeding life expectancy, especially those ≥85 years.

The indication for EVAR in younger patients remains controversial due to concerns about inferior long-term survival rates. However, one observational research by Onohara et al. reported that EVAR was not significantly associated with aneurysm-related mortality or major aneurysm-related events in 644 Japanese patients aged <70 years who underwent either open or endovascular AAA repair.^10)^ Another retrospective study reported that, in a cohort of 553 patients younger than 70 years, EVAR required more reinterventions when compared to open repair, but their long-term survival was comparable between the two groups.^11)^ Further prospective randomized studies with large sample sizes are needed to validate and develop these results.

Current guidelines from Society for Vascular Surgery,^12)^ European Society for Vascular Surgery,^13)^ and Japanese Circulation Society^7)^ do not specify indications for EVAR based on age, although patients’ life expectancy should be considered. Adjusting these guidelines to include age as a key criterion may help ensure that elderly patients receive treatment consistent with their health status and expected outcomes. Especially, in a super-aging society like Japan,^2)^ adjusting guidelines based on age could improve quality of life and optimize healthcare resources by applying EVAR to patients who are most likely to benefit from its effects.

Comorbidities have been also reported to play an important role in the long-term survival of patients undergoing EVAR.^14,15)^ This study detected age ≥85 years as well as COPD, CKD, active cancer, and non-obesity status emerged as significant adverse predictors of mortality, underscoring the impact of these conditions on physiological reserve and recovery potential. Of note is the association between non-obesity status and poor prognosis, often described as the “obesity paradox,” which may indicate that older patients with better physiological reserve have a survival advantage in cardiovascular interventions.^16,17)^ These findings suggest that adjusting the indication for EVAR based on age, life expectancy, and comorbidity burden may help avoid overtreating and optimize outcomes.

One important consideration is that the benefit of EVAR for elderly patients with smaller aneurysms (<5.5 cm) may be limited.^18,19)^ Vaughan-Burleigh et al. similarly reported that most octogenarians with AAAs near the threshold may not benefit from EVAR due to their limited life expectancy and the low risk of rupture associated with conservative management.^5)^ Our data also show that patients aged <75 years and 75–84 years underwent EVAR for small aneurysms more frequently than those aged ≥85 years, supporting the notion that conservative management may be a viable option for super elderly patients with asymptomatic, smaller aneurysms,^20)^ provided that the expected survival does not exceed the rupture risk window.

### Study limitations

This study has several limitations that should be considered when interpreting the findings. First, the data were obtained retrospectively from a prospectively maintained single-center database, which may limit the generalizability of results across diverse healthcare settings or populations. Second, our cohort was relatively small, particularly, which may reduce the statistical power to detect differences across age patient groups and may limit the precision of survival estimates. Third, this study lacks comprehensive data on frailty or functional status, factors that are particularly relevant in assessing surgical risk and potential outcomes in elderly populations.^21)^ Future multicenter studies with larger sample sizes and comprehensive frailty assessments are needed to validate and expand these findings. Fourth, we accounted for major comorbidities such as COPD and CKD, whereas no data on other potentially influential factors (e.g., cognitive status,^22)^ social support) were collected. Finally, the follow-up rate was slightly affected by the COVID-19 pandemic.

## Conclusions

This study demonstrates a cautious and individualized approach to EVAR in Japanese elderly patients. Our findings suggest that the long-term survival benefit of EVAR may be limited in the elderly, especially in the very elderly (≥85 years). In a super-aging society such as Japan, adjusting guidelines to incorporate age could improve quality of life and optimize healthcare resource allocation by focusing EVAR on patients most likely to benefit.

## Declarations

### Ethics approval statement

This study was approved by the clinical research committee of Tokyo Medical University, where it was performed (TS2020-0388, January 15, 2021). At the initial consultation, all the patients were asked whether they were willing to provide written informed consent for their clinical data to be used for scientific presentations or publications.

### Funding

This research has not received any funding.

### Data availability statement

The data that support the findings of this study are not publicly available due to confidentiality restrictions but are available from the corresponding author upon reasonable request.

### Author contributions

All authors have read and approved the manuscript, and they are responsible for the manuscript.

Nishibe T: conception and design.

Nishibe T and Kano M: analysis and interpretation.

Nishibe T, Kano M, Akiyama S, Iwahashi T, and Fukuda S: data collection.

Nishibe T: writing the article.

Nishibe T: critical revision of the article.

### Conflicts of interest

The authors have no conflicts of interest to declare.
